# Highlight: Pearls of Wisdom into Longer Lifespans From Bivalves

**DOI:** 10.1093/gbe/evad200

**Published:** 2023-11-15

**Authors:** Casey McGrath

For centuries, humans pursued the “fountain of youth” in the quest for longer lives. More recently, curiosity has been reignited among the scientific community thanks to genome sequencing technologies that are facilitating a deeper look into the genetic mechanisms underlying aging and extended lifespans. In a new study published in *Genome Biology and Evolution* titled “https://doi.org/10.1093/gbe/evad159”, researchers from the University of Bologna turned their attention to an unlikely group of creatures—bivalve molluscs, a group that includes clams, mussels, oysters, and scallops (Iannello et al. 2023). These marine and freshwater animals exhibit an astonishing range of lifespans, from 1 year to over 500 years, making them ideal subjects for investigating the secrets of longevity. The results of the new study revealed a network of genes that evolve differently in long-lived and short-lived bivalves, many of which are associated with longevity in other animals. The analyses here suggest a shared molecular framework for extended longevity across diverse animal lineages.

Prior studies on aging, longevity, and senescence have largely focused on humans and a few model animals. According to these studies, aging is largely driven by the accumulation of cellular damage over time. At the genomic level, this damage is due to increased mutations in nucleic acids (i.e., errors in replication), nuclear architecture changes, and telomere shortening. At the proteomic level, these processes result in the loss of proteases and the accumulation of errors that affect protein folding. Unfortunately, studies on aging have largely overlooked other long-lived organisms, an oversight that co-first authors Mariangela Iannello, Giobbe Forni, and their collaborators sought to rectify. “It always fascinated me that some bivalve species live extremely long lives,” says Iannello. “When I realized that nobody had ever investigated this exceptional longevity within a molecular evolution framework, I knew that we had to start studying longevity in these animals.”

The researchers leveraged transcriptomic resources from 33 bivalve species to investigate potential mechanisms underlying the exceptionally long lifespans of four bivalves: *Arctica islandica*, *Margaritifera margaritifera*, *Elliptio complanata*, and *Lampsilis siliquoidea*. Among these, the ocean quahog *A. islandica* holds the record for the longest-lived noncolonial animal species at 507 years, while the others have maximum lifespans of 150–190 years.

Using this dataset, the scientists looked for genes that evolved differently—in terms of evolutionary rate, amino acid substitutions, and signatures of positive selection—in long-lived bivalves compared to short-lived ones. Genes related to the DNA damage response, regulation of cell death and apoptotic pathways, cellular responses to abiotic stimuli, and hypoxia tolerance all showed convergent patterns of evolution across long-lived species. Intriguingly, proteins exhibiting convergent evolution in long-lived bivalves exhibited more physical and functional interactions with each other than expected, suggesting that they are biologically connected.

For many proteins in this interaction network, experimental studies have already demonstrated a role in longevity and senescence in other animals. “What I find the most exciting,” says Iannello, “is that many genes in this network had been previously associated with longevity in other species. An important implication of this finding is that an extension of lifespan may involve common genetic factors in very distantly related species.”

In addition to these shared drivers of longevity, the study identified proteins in the network whose roles in longevity have not yet been confirmed. For example, three genes involved in proteostasis (i.e., the folding, chaperoning, and maintenance of protein function) were identified, suggesting that more efficient handling of damaged or misfolded proteins may be associated with longer lives in bivalves. Iannello notes, “We believe that these genes are new and exciting candidates to be tested for a role in increasing lifespan, not only in bivalves but also in other species.”

The study authors plan to continue building on these findings through additional comparative research. According to Iannello, “The results obtained in this work made us thrilled to explore longevity in more species. In particular, we would like to investigate if the evolutionary signals in genes with a potential role in longevity are somehow shared across long-lived species from different taxonomic groups.” Such investigations may not be straightforward, however. “A complex and multifactorial process such as longevity is definitely challenging to analyze, requiring deep manipulation of big data and multiple complementary, integrative approaches,” says Iannello. “On the other hand, the increasing availability of omics data will allow us to explore species that have never been considered in this context before, and that would greatly help advance aging research.”

While the mechanisms that underlie extended lifespan remain far from completely understood, long-lived nonmodel organisms can provide unique and valuable insights into aging and longevity. For example, in another recent study from *Genome Biology and Evolution*, researchers from University College Dublin analyzed genes associated with human longevity across 37 placental mammals, including long-lived species such as the naked mole rat and the greater mouse-eared bat (Huang et al. 2023). The study found a correlation between longer lifespans and the duplication of longevity genes. While some may be skeptical about transferring knowledge across very distant species, such as between bivalves and humans, Iannello points out, “Science has a long history of research focused on the most disparate taxa that has profoundly impacted our understanding of human biology. I think that, particularly in the aging field, we have a lot to learn from the natural world around us.”
